# The association between social camouflage and mental health among autistic people in Japan and the UK: a cross-cultural study

**DOI:** 10.1186/s13229-023-00579-w

**Published:** 2024-01-04

**Authors:** Fumiyo Oshima, Toru Takahashi, Masaki Tamura, Siqing Guan, Mikuko Seto, Laura Hull, William Mandy, Kenji Tsuchiya, Eiji Shimizu

**Affiliations:** 1 Research Center for Child Mental Development, Chiba University, 1-8-1 Inohana, Chuouku, Chiba 260-8670 Japan; 2 United Graduate School of Child Development, Osaka University, Kanazawa University; Hamamatsu University School of Medicine, Chiba University, and University of Fukui, Osaka, Japan; 3 Laureate Institute for Brain Research, Tulsa, OK USA; 4 Japan Society for the Promotion of Science, Tokyo, Japan; 5 Department of Cognitive Behavioral Physiology, Graduate School of Medicine, Chiba University, Chiba, Japan; 6 Graduate School of Human Sciences, Waseda University, Tokorozawa, Saitama Japan; 7 Research Department for Clinical, Educational & Health Psychology, University College London, London, UK; 8 Research Center for Child Mental Development, Hamamatsu University School of Medicine, Hamamatsu, Japan; 9 Centre for Academic Mental Health, Department of Population Health Sciences, Bristol Medical School, University of Bristol, Bristol, UK

**Keywords:** Autistic adults, Social camouflage, Cross-cultural study, Mental health, Japan, UK

## Abstract

**Background:**

To examine the relationship between social camouflage and mental health in Japanese autistic adults and make an international comparison with a sample from the UK.

**Methods:**

This study analysed secondary data of participants with a self-reported diagnosis of autism from Japan (N = 210; 123 men and 87 women) and the UK (N = 305; 181 women, 104, men, and 18 nonbinary). The relationships between the quadratic term of the Camouflaging Autistic Traits Questionnaire and mental health scales, including depression and anxiety, were assessed.

**Results:**

The UK sample showed linear relationships, whereas the Japanese sample showed significant nonlinear relationships. The quadratic terms of the Camouflaging Autistic Traits Questionnaire slightly explained generalised anxiety (β = .168, *p* = .007), depression (β = .121, *p* = .045), and well-being (β = − .127, *p* = .028). However, they did not explain the association between social anxiety and the Camouflaging Autistic Traits Questionnaire.

**Limitations:**

Participants had self-reported diagnoses, and while the autism-spectrum quotient provides a cut-off value for screening, it does not enable confirming diagnoses. Mean scores of the Japanese version of the Camouflaging Autistic Traits Questionnaire were lower as compared to the original CAT-Q, which implies that the social camouflage strategy types used by autistic people in Japan and the UK could differ. The cross-sectional design limits causal inferences.

**Conclusion:**

In the UK, more social camouflage was associated with poorer mental health scores, whereas too little or too much social camouflage was associated with a low mental health score in Japan. The Japanese population is seemingly less aware of and educated on autistic characteristics and considers ‘average’ behaviour a good thing. This could influence Japanese autistic people’s social camouflage use, differing from that of autistic people in the UK. The differences in the relationship between social camouflage and mental health between Japan and the UK could be associated with national-level divergence regarding the culture of autism.

**Supplementary Information:**

The online version contains supplementary material available at 10.1186/s13229-023-00579-w.

## Background

Autistic people tend to show differences in social communication and interaction; they tend to express a focus on specific interests and are sensitive to sensory stimulation [1]. Although autism can be identified in infancy, it can also be diagnosed across people’s lifespan, in turn helping them identify their specific strengths and needs. Importantly, many autistic people are not diagnosed until adulthood [2, 3]. This study uses the terms autistic people and autism, not autism spectrum disorder, to avoid ableist language [4].

As autistic individuals grow into adults, mental health issues account for a more significant percentage of the symptoms that they experience than the core symptoms of autism [5–7]. Researchers have reported that 79% of autistic adults have mental health problems, such as depression, anxiety, social anxiety, and obsessive–compulsive symptoms [8, 9]. In addition to these psychiatric disorders, almost all medical conditions were significantly more common in autistic adults, including immune disorders, gastrointestinal disorders, sleep disorders, seizures, obesity, dyslipidaemia, hypertension, and diabetes [10]. Although autism is not a mental health problem, autistic people could experience functional impairments resulting from autistic traits and the aforementioned mental health conditions, which together could lead to an increased risk of withdrawal from employment, suicide, self-harm, and low quality of life [11–14]. Therefore, identifying the link between autism and mental health is crucial to promoting mental health prevention in this population.

Although data on the mental health of autistic people in Japan are limited, autistic children who receive support in early childhood (age 10 years or younger) have better mental health outcomes in adulthood than those who do not receive support [15]. A 20 year follow-up study of individuals diagnosed with autism in early childhood found that approximately 40% of them had a good quality of life later on in life [16]. These pieces of evidence indicate that mental health maintenance in autistic individuals is associated with support from an early age.

Social camouflage refers to the efforts by autistic people to consciously or unconsciously modify their behaviour to fit in with non-autistic people [2]. It also refers to strategies that they use, either consciously or unconsciously, to conceal their autistic characteristics and compensate for the social difficulties associated with autism [7]. As many as 70% of autistic individuals are aware that they engage in social camouflage and ‘have to’ do so [17, 18]. Social camouflage is described as having the three elements of ‘compensation’ (i.e. strategies to overcome social difficulties associated with autistic traits), ‘masking’ (i.e. strategies to hide one’s autistic traits), and ‘assimilation’ (i.e. strategies to blend in with others in social situations [19]). Many autistic individuals engage in social camouflage and attempt to use social interaction to obtain job opportunities and other benefits. The aforementioned ‘need’ of autistic individuals to engage in social camouflage forces them to continuously pretend that they are non-autistic. This is associated with significant manifestations of mental health deterioration, such as depression, generalised anxiety, social anxiety [20, 21], suicide attempts, and burnout because of exhaustion and fatigue [22].

Although one of the reasons autistic individuals are motivated to engage in social camouflage is a desire to fit in with society, some believe they engage in such behaviours as a response to social stigma. Stigma can be found in various forms in society, and one’s perception of the social stigma of autism is a significant sociopsychological factor contributing to mental health deterioration in autistic adults [23]. Autistic teenagers feel ashamed if they perceive that they are bothering others, which reduces their willingness to achieve reasonable adaptation [22]. Social stigma is more prevalent in Asian than in Western countries. For example, some researchers have reported greater knowledge, familiarity, and acceptance of autism in the UK than in Malaysia [24]; some have also reported that South Koreans showed more stigma toward autism than Americans [25]. Furthermore, while 38% of the Chinese participants of a study endorsed stigma toward autism, only 14% of the American participants did so [26], and American students showed less stigma toward autism than Lebanese students [27]. In Japan, Japanese students showed more stigma toward autism than American students [28]. Markus and Kitayama [29] refer to Japanese and other East Asian cultures as ‘cultures of interdependence’. In these cultures, the primary challenge faced by individuals is to conform without standing out and pay more attention to others than oneself. Thus, the ‘uniqueness’ of autistic people can be perceived negatively, and it can threaten relationships and interpersonal harmony within the community. Japan has an interdependent culture, and researchers have indeed described that being ‘unique’ in Japan is often seen as a ‘threat’ [28]. These delineations led us to consider the possibility that the culture of interdependence could force Japanese autistic individuals to over-adapt or camouflage to non-autistic groups.

As aforementioned, one of the key motivations for autistic people to engage in social camouflage is their desire to fit in with other people, make a good impression, make friends, and get to know others better [2, 30, 31]. Another motivation that autistic adults identified for engaging in social camouflage was to avoid adverse reactions from other people for looking autistic [2, 30, 31]. These motivations are similar to non-autistic individuals’ for using impression-management strategies. However, autistic people might require more effort to use these strategies, use them in more situations, and use them for a longer period than non-autistic individuals [19]. Another study showed that the perception of stigma toward autism promotes social camouflage among autistic individuals [32]. In 2022, academicians investigated the relationship between the perception of stigma toward autism and social camouflage in Japan [33]. Their results were similar to those of another research [32] finding that autistic individuals with a higher social stigma perception engaged more in social camouflage. Therefore, autistic people’s perceptions of social stigma toward autism contribute to their greater engagement in social camouflage in both the UK and Japan.

If autistic people use social camouflage as a strategy for dealing with the stigma toward autism, it could be reasonable to expect that a reduction in social stigma leads to a lower need to engage in social camouflage. Cage et al. [34] also noted that feeling accepted by others as an autistic person could be a protective factor against depression [34]. However, researchers investigating social camouflage and mental health have mostly referred to the outcomes of this association of autistic people based only on Western culture. Reducing social stigma against autism reduces the social camouflage of autistic individuals, and the amount of social camouflage that autistic individuals engage in could vary by country because the social stigma of autism varies by culture [27, 28, 35]. Given that the social stigma experienced by autistic people varies from culture to culture, the difference between social camouflage and mental health problems is also expected to differ from culture to culture. Still, to date, there is no identification.

In Japan, as noted above, few studies have examined the association between autistic adults and mental health. Among the few examples, the studies by Kamio et al. [15] and Iwasa [16] examined the quality of life of Japanese autistic adults but not their mental health. Moreover, to our knowledge, the influence of social camouflage on mental health deterioration among autistic people in Asian countries, such as Japan, has yet to be explored. The situation of Japanese autistic adults, whose psychosocial quality of life tends to be lower than that of the general Japanese adult population [15], is similar to that of autistic adults in Western Europe. However, past studies conducted with Japanese autistic adults have focused mostly on the link between poorer mental health and backward-looking factors (e.g. IQ, the timing of diagnosis, and maternal support during childhood [15, 16]), and none thus far has looked at the association between mental health and social camouflage in Japanese autistic individuals. Since different cultural backgrounds can influence the relationship of these variables and the aforementioned studies corroborate this possibility, there could be differences between Eastern and Western societies for the association of mental health and social camouflage among autistic individuals. Therefore, we considered it worthwhile to make an international comparison of social camouflage and its influence on various psychosocial factors of autistic people.

## Study aims

This study is a replication and re-analysis of the study by Hull et al. [20], which investigated autistic adults in the UK; it aims to clarify the characteristics of Japanese autistic people regarding social camouflage and mental health, as well as compare them with those of autistic people in the UK. In Hull et al. [20], the scholars observed a linear correlation between social camouflage and mental health issues (i.e. depression, social anxiety, and general anxiety) in autistic people. However, we hypothesised that too much or too little engagement in social camouflage would negatively affect the mental health of autistic people in Japan. Given this cultural context and the tendency toward negative reactions to the ‘differences’ of autism, we hypothesised that the relationship between social camouflage and mental health is quadratic. Hull et al. [20] did not consider the possibility that either too much or too little engagement in social camouflage could negatively affect mental health. Accordingly, while they did consider a monotonically increasing quadratic relationship, we not only used the methodology present in Hull et al.’s [20] investigation but also probed into whether the following posit is true: the longer the distance between the score and the average, the poorer the mental health. That is, we checked for the existence of a quadratic curve for the analysed association. The current research also analysed the relationship between sex and mental health in Japanese and British autistic adults.

## Methods

### Participants

This study conducted secondary analyses of the data from a past study conducted to standardise the Japanese version of the Camouflaging Autistic Traits Questionnaire (CAT-Q; Hongo et al. [36]). To compare data from Japanese and UK samples, we used the dataset of Hull et al. [20] study, with a sample of adults with a self-reported diagnosis of autism in the UK (N = 305, aged 18–75 years), regarding scores for the CAT-Q and other mental health questionnaires. We requested the authors for access to their dataset and approval for its use in research, which they approved. In Hongo et al.’s [36] study, which was the main subject of our investigation, participants were 210 Japanese autistic men and women. Although Hongo et al. [36] excluded data from six participants who consistently gave the same response for scales (i.e. straight liners) that included reversed items, we used data from all participants following the procedures in Hull et al. [20] because responses from straight liners cannot necessarily be regarded as invalid responses [37]. The results of the analyses while excluding straight liners are shown in Additional file 1.

The descriptive statistics of participants in Japan are presented in Table 1. The questionnaire in Hull et al. [20] study allowed participants to respond that they were of nonbinary gender, whereas the data of Hongo et al. [36] study only contained two options for sex: men and women. Of the participants in Hull et al. [20] study, 104 (34.1%) were men, 181 (59.3%) were women, and 18 (5.9%) were nonbinary. Their age ranged from 18 to 75 years (M = 41.90 years, standard deviation [SD] = 13.60), and the mean age at diagnosis was 34.89 years (SD = 14.44). Of the participants in Hongo et al. [36] study, 123 (58.6%) were men, and 87 (41.4%) were women. Their age ranged from 20 to 64 years (M = 37.53 years, standard deviation [SD] = 10.33), and the mean age at diagnosis was 28.70 years (SD = 12.16). Furthermore, 114 (55.9%) were employed full- or part-time, 21 (10.3%) were students, 11 (5.39%) were full-time homemakers, and 58 (28.4%) were unemployed or unable to work.

**Table 1:** Characteristics of Japanese participants and means and standard deviations for all variables by sex

	Total sample(N = 210)			Female subsample (n = 87)			Male subsample (n = 123)		
	Mean	SD	n	Mean	SD	n	Mean	SD	n
Age (Years)	37.61	10.31	210	38.86	10.31	87	35.84	10.05	123
Diagnosis									
Autism spectrum disorder (%)	131	(62%)		73	(59%)		58	(67%)	
Developmental disability (%)	55	(26%)		34	(28%)		21	(24%)	
Pervasive developmental disorders (%)	74	(35%)		41	(33%)		33	(38%)	
Autistic disorder (%)	38	(18%)		27	(22%)		11	(13%)	
Asperger disorder (%)	73	(35%)		41	(33%)		32	(37%)	
Attention deficit hyperactivity disorder (%)	58	(28%)		34	(28%)		24	(28%)	
Schizophrenia (%)	0	(0%)		0	(0%)		0	(0%)	
Dementia (%)	0	(0%)		0	(0%)		0	(0%)	
Intellectual disability (%)	0	(0%)		0	(0%)		0	(0%)	
Autistic traits (BAPQ)	4.00	.70	205	3.93	.67	85	4.10	.73	120
Social camouflage (CAT-Q)	105.29	21.94	209	101.20	20.48	86	111.14	22.61	123
Social anxiety (LSAS)	72.77	36.87	200	69.89	35.19	83	76.82	38.76	117
Generalised anxiety (GAD-7)	9.03	6.17	208	7.87	5.66	85	10.71	6.49	123
Depression (PHQ-9)	11.93	7.26	209	10.40	6.91	87	14.08	7.19	122
Well-being (WEMWBS)	36.25	10.22	209	37.28	9.77	87	34.80	10.65	123

*BAPQ* Broad autism phenotype questionnaire; *CAT-Q* Camouflaging Autistic Traits Questionnaire; *GAD-7* Generalized anxiety disorder assessment-7; *LSAS* Liebowitz social anxiety scale; *PHQ-9* Patient health questionnaire-9; *WEMWBS* Warwick–Edinburgh Mental Well-being Scale

The inclusion criteria were being at or above the legal age to provide informed consent on own behalf in Japan (20 years of age) and being officially diagnosed with autism: ‘autism spectrum disorder’, ‘autism’, ‘Asperger’s syndrome/disorder’, or ‘pervasive developmental disorder’, and not otherwise specified. Those who reported being self-diagnosed or diagnosed with only ‘autistic traits’ were excluded from this study.

Hongo et al. [36] recruited participants and patients attending medical institutions between February 2020 and April 2021 through online survey panels. As an honorarium, participants received 1000 yen (approximately USD 7). The Research Ethics Committee of Chiba University Research Ethics Committee (reference M10053) approved this study, and written and oral informed consent was obtained from all participants before the study onset.

### Materials and procedures

Since this study was a secondary analysis of the data of Hongo et al. [36], readers are referred to this cited study for more details on participants’ recruitment and inclusion criteria, among other methodological topics.

#### The Japanese version of the camouflaging autistic traits questionnaire

The questionnaire included the Japanese version of the CAT-Q (CAT-Q-J [36]). The CAT-Q comprises 25 items across three subscales [19], as follows: compensation (nine items), masking (eight items), and assimilation (eight items). Each item is rated on a seven-point Likert scale ranging from 1 (*strongly disagree*) to 7 (*strongly agree*). A higher total score for the CAT-Q scale indicated more severe social camouflaging. In total, 402 participants (200 autistic and 202 non-autistic) completed the CAT-Q. The CAT-Q has good sensitivity and specificity [19], and its internal consistency in Hongo et al. [36] was good (total sample, Cronbach’s α = 0.88). Although the CAT-Q has three subscales, to ensure compatibility with the methods used by Hull et al. [19], this study used only the total score.

#### The Japanese version of the liebowitz social anxiety scale

The questionnaire contained the Japanese version of the Liebowitz Social Anxiety Scale (LSAS-J), which is based on the LSAS [38]. This self-report scale comprises 48 items that measure social anxiety and two subscales with 24 items each: social fear and social avoidance. Each scale was assessed using a four-point Likert scale, as follows: social fear was rated from 0 (*no feeling at all*) to 3 (*powerful feeling*), and social avoidance was rated from 0 (*never*) to 3 (*two-thirds or 100% of avoidance probability*). Thus, the total score ranged from 0 to 144. The cut-off value for social anxiety was 44 points (sensitivity, 93.3%; specificity, 90.0%; [39]). The LSAS-J has high reliability and validity [38], and its internal consistency in Hongo et al. [36] was excellent (total sample, Cronbach’s α = 0.98).

#### The Japanese version of the Warwick–Edinburgh mental well-being scale

The questionnaire included the Japanese version of the Warwick–Edinburgh Mental Well-being scale (WEMWBS-J), which is based on the WEMWBS [40]. This 14-item self-report questionnaire measures general well-being over the last two weeks, with higher scores suggesting more positive mental well-being [41]. An example item is ‘I’ve been feeling relaxed’, and items are rated on a five-point Likert scale ranging from 1 (*none of the time*) to 5 (*all the time*). The WEMWBS has demonstrated acceptable validity and reliability [41]. The internal consistency of the WEMWBS in Hongo et al. [36] was excellent (total sample, Cronbach’s α = 0.91).

#### The Japanese version of the patient health questionnaire-9

The questionnaire included the Japanese version of the Patient Health Questionnaire-9 (PHQ-9), which is a simplified assessment tool for measuring major depressive disorder [42]. Responses were scored using a four-point scale ranging from 0 (*not at all*) to 3 (*nearly every day*), and total scores ranged from 0 to 27 points. The internal consistency of the PHQ-9 in Hongo et al. [36] was excellent (total sample, Cronbach’s α = 0.90).

#### The Japanese version of the generalized anxiety disorder-7

The questionnaire contained the Japanese version of the Generalized Anxiety Disorder-7 (GAD-7), which is a simplified version of the GAD [42]. Responses were scored using a four-point scale ranging from 0 (*not at all*) to 3 (*nearly every day*), and total scores ranged from 0 to 21 points. The internal consistency of the GAD-7 in this study (total sample) was excellent (Cronbach’s α = 0.91).

### Statistical analysis

Because data were missing for some measures of some participants, multiple imputations were performed to reduce the potential for bias by maximising the usable proportion of the sample. Multiple imputations were performed using the missing values package in SPSS (IBM, Armonk, NY, USA), with estimates from five imputations pooled to produce imputed data. The missing values were supplemented for missing scale (not item) scores, as was done in Hull et al. [20]. Bivariate correlations between all variables were calculated for the total sample (Additional file 1: Table S1). Bivariate correlations between all variables for the sample excluding straight liners are shown in (Additional file 1: Table S2).

Hongo et al. [36] conducted a replication study in Japan of the research by Hull et al. [20], allowing for data comparisons to be made. Specifically, we used hierarchical multiple regression analyses with a quadratic term for social camouflage (CAT-Q^2^) and an interaction term (camouflage*sex) to predict the mental health variables. Hull et al. [20] did not perform centring (subtracting the mean from individual scores) for the scores for CAT-Q in their study. However, we performed a centring procedure on the Japanese and UK CAT-Q data. Standardisation was performed for each scale because standardisation also serves as a centring procedure. All variables were standardised before being entered into the multiple regression models to show the standardised partial regression coefficients. The significance level was set at 5%.

## Results

Is there a quadratic relationship between social camouflage and mental health problems, such as generalised anxiety, depression, and social anxiety that is related to social camouflage in autistic individuals?

**Fig. 1:**
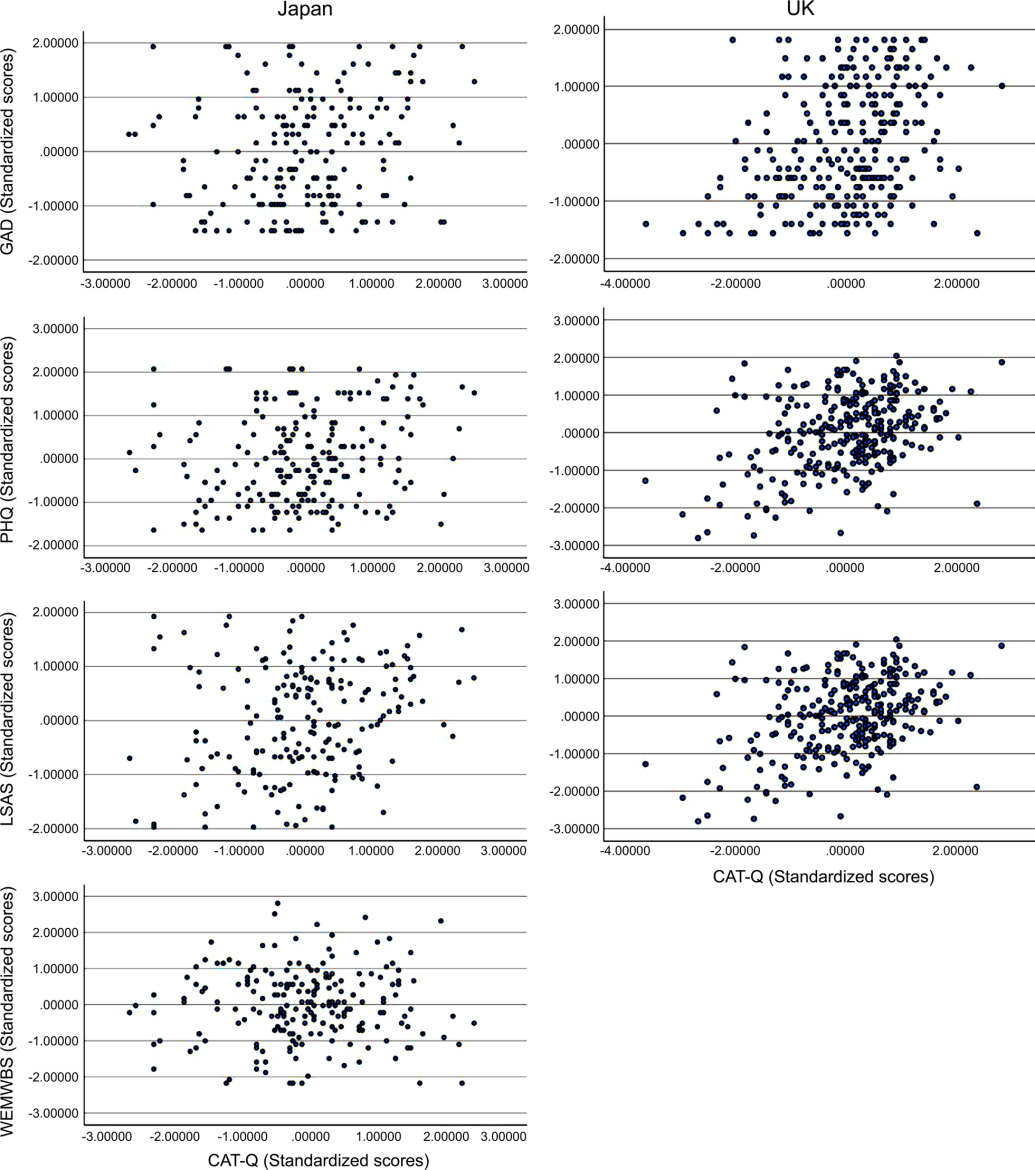
Scatter plots between standardised scores for generalised anxiety, depression, social anxiety, well-being, and social camouflage in the Japanese and UK samples. CAT-Q, Camouflaging Autistic Traits Questionnaire; GAD, Generalized Anxiety Disorder-7; PHQ, Patient Health Questionnaire-9; LSAS, Liebowitz Social Anxiety Scale; WEMWBS, Warwick–Edinburgh Mental Well-being Scale

The UK data did not show nonlinear relationships, whereas the Japanese data showed significant nonlinear relationships. The results are presented in Table 2, where the quadratic terms of the CAT-Q-J slightly explained generalised anxiety (GAD-7; β = 0.168, *p* = 0.007), depression (PHQ-9; β = 0.121, *p* = 0.045), and well-being (WEMWBS-J; β = − 0.127, *p* = 0.028). However, the quadratic terms did not explain the association between social anxiety (LSAS-J) and the CAT-Q-J. The results of these analyses, excluding straight liners, are presented in Additional file 1: Table S3. Scatter plots (before multiple assignments between the CAT-Q-J and other variables) are shown in Fig. 1 (Scatter plots excluding straight liners are shown in Additional file 1: Fig. S1).

**Table 2:** Hierarchical regression models predicting generalised anxiety (Model 1), depression (Model 2), social anxiety (Model 3), and well-being (Model 4) according to age, autistic traits, and social camouflage in the Japanese and UK samples

		Japan (N = 210)				UK (N = 305)			
	Variable	β	*p*	95% CI		β	*p*	95% CI	
				Lower	Upper			Lower	Upper
Model 1									
(Generalised anxiety)									
Step 1									
	Age	0.053	0.403	− 0.071	0.177	− 0.129	0.059	− 0.245	− 0.013
	BAPQ	0.423	< 0.001	0.299	0.547	0.399	0.054	0.292	0.506
Step 2									
	Age	0.066	0.321	− 0.064	0.195	− 0.089	0.060	− 0.210	0.032
	BAPQ	0.410	< 0.001	0.281	0.539	0.347	0.056	0.237	0.457
	CAT-Q	0.050	0.475	− 0.087	0.186	0.219	0.061	0.098	0.340
Step 3									
	Age	0.072	0.267	− 0.055	0.200	− 0.089	0.060	− 0.210	0.032
	BAPQ	0.411	< 0.001	0.284	0.539	0.346	0.056	0.236	0.457
	CAT-Q	0.068	0.325	− 0.067	0.203	0.217	0.064	0.090	0.345
	CAT-Q^2^	0.168	0.007	0.045	0.290	− 0.005	0.056	− 0.115	0.104
Model 2									
(Depression)									
Step 1									
	Age	0.074	0.229	− 0.046	0.193	− 0.151	0.058	− .0266	− 0.036
	BAPQ	0.488	< 0.001	0.368	0.608	0.370	0.054	0.265	0.476
Step2									
	Age	0.092	0.145	− 0.032	0.216	− 0.129	0.061	− 0.250	−0.008
	BAPQ	0.469	< 0.001	0.345	0.593	0.342	0.055	0.233	0.450
	CAT-Q	0.073	0.265	− 0.055	0.201	0.122	0.062	− 0.003	0.246
Step 3									
	Age	0.097	0.122	− 0.026	0.220	− 0.129	0.061	− .0250	− 0.008
	BAPQ	0.470	< 0.001	0.347	0.593	0.341	0.056	0.232	0.450
	CAT-Q	0.086	0.187	− 0.042	0.214	0.121	0.066	− 0.011	0.252
	CAT-Q^2^	0.121	0.045	0.002	0.240	− 0.004	0.058	− 0.118	0.110
Model 3									
(Social anxiety)									
Step 1									
	Age	0.062	0.289	− 0.053	0.177	− 0.116	0.048	− .210	− 0.022
	BAPQ	0.592	< 0.001	0.481	0.704	0.600	0.047	0.508	0.692
Step 2									
	Age	0.075	0.228	− 0.047	0.196	− 0.076	0.047	− 0.168	0.017
	BAPQ	0.580	< 0.001	0.463	0.697	0.547	0.047	0.454	0.640
	CAT-Q	0.048	0.437	− 0.074	0.171	0.224	0.048	0.130	0.319
Step 3									
	Age	0.077	0.211	− 0.044	0.199	− .075	0.047	− 0.168	0.018
	BAPQ	0.580	< 0.001	0.463	0.698	0.545	0.047	0.452	0.638
	CAT-Q	0.056	0.372	− 0.067	0.179	0.219	0.052	0.117	0.322
	CAT-Q^2^	0.070	0.226	− 0.043	0.183	− 0.017	0.054	− 0.124	0.089
Model 4									
(Well-being)									
Step 1									
	Age	− 0.088	0.137	− 0.204	0.028				
	BAPQ	− 0.528	< 0.001	− 0.644	− 0.412				
Step 2									
	Age	− 0.046	0.443	− 0.165	0.072				
	BAPQ	− 0.570	< 0.001	− 0.690	− 0.451				
	CAT-Q	0.163	0.010	0.039	0.286				
Step 3									
	Age	− 0.052	0.389	− 0.169	0.066				
	BAPQ	− 0.571	<0 .0001	− 0.690	− 0.453				
	CAT-Q	0.149	0.018	0.026	0.272				
	CAT-Q^2^	− 0.127	0.028	− 0.241	− 0.014				

*BAPQ* Broad autism phenotype questionnaire; *CAT-Q* Camouflaging Autistic Traits Questionnaire; *β* Standardised beta; *CI* Confidence interval. In the UK sample, well-being was not measured. The quadratic term of standardised social camouflage scores (CAT-Q^2^) was added in Step 3

### Is the relationship between mental health and social camouflage restrained by sex?

To examine the influence of sex on the relationship between social camouflage and mental health, hierarchical linear regression analyses were run with the binary variable of sex (men and women) as a baseline predictor (alongside age, autistic traits, and social camouflage) in the first stage. Then, an interaction term (camouflage*sex) was added to the models in the second stage. The interactions between sex and social camouflage were not significant in any model (Table 3). The results of these analyses excluding straight liners are presented in Additional file 1: Table S4.

**Table 3:** Hierarchical regression models predicting generalised anxiety (Model 1a), depression (Model 2a), social anxiety (Model 3a), and well-being (Model 4a) according to age, sex, autistic traits, and social camouflage in the Japanese sample (N = 210)

	Variable	β	*p*	95% CI	
				Lower	Upper
Model 1a					
(Generalised anxiety)					
Step 1					
	Age	0.083	0.201	− 0.044	0.211
	Sex	0.190	0.003	0.064	0.316
	BAPQ	0.396	< .0001	0.269	0.523
	CAT-Q	0.015	0.823	− 0.120	0.151
Step 2					
	Age	0.083	0.202	− 0.045	0.211
	Sex	0.190	0.003	0.064	0.316
	BAPQ	0.396	< 0.001	0.269	0.524
	CAT-Q	0.016	0.821	− 0.120	0.152
	CAT-Q*Sex	− 0.002	0.970	− 0.127	0.122
Model 2a					
(Depression)					
Step 1					
	Age	0.110	0.077	− 0.012	0.232
	Sex	0.194	0.002	0.073	0.314
	BAPQ	0.455	< 0.001	0.333	0.577
	CAT-Q	0.038	0.559	− 0.090	0.166
Step 2					
	Age	0.110	0.077	− 0.012	0.232
	Sex	0.195	0.002	0.074	0.316
	BAPQ	0.453	< 0.001	0.331	0.576
	CAT-Q	0.041	0.534	− 0.088	0.169
	CAT-Q*Sex	− 0.024	0.696	− 0.142	0.095
Model 3a					
(Social anxiety)					
Step 1					
	Age	0.078	0.209	− 0.044	0.199
	Sex	0.033	0.593	− 0.090	0.157
	BAPQ	0.577	< 0.001	0.460	0.695
	CAT-Q	0.043	0.502	− 0.082	0.167
Step 2					
	Age	0.078	0.207	− 0.043	0.200
	Sex	0.037	0.558	− 0.087	0.160
	BAPQ	0.574	< 0.001	0.456	0.692
	CAT-Q	0.049	0.442	− .0076	0.174
	CAT-Q*Sex	− 0 .053	0.354	− 0.165	0.059
Model 4a					
(Well-being)					
Step 1					
	Age	− 0 .055	0.360	− 0.174	0.063
	Sex	− 0.097	0.107	− 0.214	0.021
	BAPQ	− 0.563	< 0.001	− 0.682	− .0444
	CAT-Q	0.180	0.005	0.055	0.305
Step 2					
	Age	− 0.055	0.364	− 0.173	0.063
	Sex	− 0 .092	0.125	− 0.209	0.026
	BAPQ	− 0.569	< 0.001	− 0.688	− 0.450
	CAT-Q	0.190	0.003	0.065	0.315
	CAT-Q*Sex	− .0080	0.178	− 0.196	0.036

*BAPQ* Broad autism phenotype questionnaire; *CAT-Q* Camouflaging autistic traits questionnaire; *β* Standardised beta; *CI* Confidence interval

## Discussion

This study aimed to clarify the characteristics of Japanese autistic people regarding social camouflage and mental health and compare them with those of autistic people in the UK. It showed that too much or too little engagement in social camouflage would negatively affect the mental health of autistic people in Japan, as we hypothesised. In the hierarchical multiple regression analysis, the Japanese dataset showed nonlinear results (i.e. too little or too much social camouflage was related to more severe mental problems), whereas the UK dataset showed linear results. In the UK, more social camouflage was associated with poorer mental health, while too little or too much social camouflage was associated with poorer mental health in Japan.

The average engagement in social camouflage among Japanese autistic people was lower than that among autistic people in the UK.

In the Autism Knowledge Quiz [43], which conducted a randomised controlled trial to quantify autism awareness, Japanese autistic adolescents had a lower self-understanding of the autism spectrum disorder than British autistic adolescents. In addition, while the cut-off for the autism-spectrum quotient [44] is 33 points in the UK, the cut-off is 26 points in Japan [45]. This means that autistic people in Japan have few opportunities to receive education about autistic traits and have difficulty understanding what autistic traits are, causing them to feel that they do not fit in with society. For example, instead of the term social camouflage, a similar concept exists in Japan, i.e. autistic people over-adapt. This is considered to be synonymous with social camouflage in terms of the content. Relatedly, in an international comparative study conducted in 2023, autistic individuals in Japan were found to be overadjusted in eight countries (Australia, Belgium, Canada, Japan, New Zealand, South Africa, the United Kingdom, and the United States), and Japanese autistic individuals had both the lowest social acceptance and the lowest internal acceptance scores [46]. This indicates a high external pressure in Japan to be like normal people [46]. Japan is an East Asian country with a relatively collectivistic and homogeneous culture and high cultural density [28, 47], and Japanese society has a particularly high stigma against autistic individuals because they sometimes deviate from social norms. For example, compared to the UK and India, a stronger emphasis is placed on social conformity in Japanese society [48].

Japanese people tend to be intolerant of ‘people who look different’, such as autistic people. However, it is also rare for Japanese people to point this out to Japanese autistic people because people in the general population tend to be nonverbal toward autistic people. Therefore, autistic people in the country do not have many opportunities to become aware of their autistic traits. Consequently, strategies for adaptation could manifest as over-adaptation in Japanese autistic adults because they could end up preferring to ‘follow one’s orders’ rather than engage in social camouflage, which is an impression-management strategy that requires some understanding of autistic traits.

Another reason for the results of a reduced engagement in social camouflage among Japanese autistic adults could be related to the fact that regarding social orientation, people in Asia are less independent and more interdependent than people in Western countries [29]. As mentioned in the Introduction section, the term interdependence refers to the emphasis on remaining harmonious with a homogeneous group, and many Asian cultures have a culture of interdependence [29]. Western cultures generally do not assume overt ties between individuals and do not recognise the value of interdependence as much as Asian cultures. High levels of interdependence lead to high levels of cohesion between groups, thus also leading to the exclusion of groups with attributes that deviate from shared moral boundaries [49]. Based on these descriptions, we hypothesised that living in a culture characterised by interdependence could be related to moderate levels of engagement in social camouflage, having a positive influence on mental health among autistic individuals. Moderate levels of social camouflage were associated with moderate levels of mental health, specifically in Japanese autistic adults. This is consistent with the reports that both autistic and non-autistic Japanese people engage less in social camouflage than their British counterparts [36].

### The relationship between social stigma and social camouflage

In both Japan and the UK, perceived social stigma was one of the factors associated with social camouflage; however, a study showed that the prevalence of social stigma among non-autistic people was greater in Asian than in Western countries [28]. At first glance, these results for perceived social stigma and actual social stigma could appear contradictory. Nonetheless, our results showed that the perception of social stigma of autistic people in Japan is lower than that of autistic people in the UK. Therefore, there could be a dissociation between the actual existence and the perception of social stigma in Japan. While the average age of autism diagnosis in 40 countries is 60.48 months (range: 30.90–234.57 months [50]), the average age of diagnosis in Japan is approximately 10 years (120 months [15]), which shows a delay in the diagnosis in Japan. This suggests that Japanese society has little awareness or understanding of autism. Thus, the lower social camouflage of Japanese autistic people than that of autistic people in the UK could be partly because Japanese autistic people are unaware of their autistic characteristics owing to the lack of educational opportunities for autism.

### The influence of sex on the relationship between mental health problems and social camouflage

The relationship between social camouflage and mental health problems was not linear in Japan, different from that in the UK. One of the reasons could be the predominance of collectivism in Japan, making it so that most social behaviours of Japanese people are largely determined by goals, attitudes, and values that are shared with a group of people [51]. Non-autistic Japanese people also seem to have fewer opportunities to receive education on, and have less understanding of, autistic traits at both the social and individual levels [28]. This could mean that they feel more secure by behaving in a more ‘discreet’ manner, as this could avoid social exposure, rather than behaving more in line with their specific traits. However, such repressive attempts to assimilate into the group may be related to poor mental health, depending on the extent of such efforts. Alongside these influences, gender role expectations in the Japanese culture could also be related to how autistic adults engage in social camouflage—as well as the association of the latter with mental health.

Our research indicates that like Hull et al. [20] study, the link between social camouflage and mental health (such as generalised anxiety, depression, and social anxiety) remains consistent across sexes among autistic adults. However, some scholars have found that autistic women tend to engage in social camouflage more so than autistic men [52]. There is some evidence suggesting that social camouflage could be influenced by sex and the time of diagnosis [53], although the reasons for these variations are not yet fully understood. Some believe that gender role expectations play a role in encouraging social camouflage. For instance, traditional gender roles in Japan (such as women being expected to be quiet, do household chores, and take care of children, while men are expected to earn money and work hard) put different social pressures on each gender [54]. Although these expectations are divergent, they could still motivate both sexes to engage in social camouflage, which could explain why there was no interaction effect for both sexes among Japanese autistic adults. However, the link between sex and social camouflage requires further research, as there are no previous studies in Japan.

Furthermore, Hull et al. [20] argued that research examining sex in the context of social camouflage and mental health needs to specifically address the experiences of people with nonbinary gender identity. In the Japanese survey, there was no item for people to express their gender as being nonbinary, whereas the survey conducted in the UK allowed people to express their gender, and a small number of people responded that they were nonbinary. Nonetheless, the sample size for these nonbinary respondents was not sufficient to enable statistical comparisons to be made. Correlation analysis of mental health problems and social camouflage in the nonbinary subsample was also not significant. Notwithstanding, significant associations could be identified in larger properly sized samples and should be conducted in Japan in the future.

### Limitations

This study has several limitations. First, the autistic participants had self-reported diagnoses, and while the autism-spectrum quotient provides a cut-off value for screening, it does not enable confirming diagnoses. Second, the mean score for the CAT-Q-J was lower than that for the original CAT-Q [55]. This implies that the social camouflage strategy types used by autistic people in Japan and the UK could differ, albeit this study does not examine this topic. This could be further explored in future studies. Finally, although this study conducted a hierarchical multiple regression analysis, it was cross-sectional and did not enable examining causal relationships. In the future, researchers could conduct a study using longitudinal data with the variables of age and time to clarify the causal relationship between social camouflage and mental health among autistic Japanese individuals.

## Conclusion

In Japanese autistic adults, the association between social camouflage and mental health problems was non-linear, with moderate social camouflage being positively associated with mental health. However, excessive social camouflage was found to worsen mental health. This can be attributed to multiple factors, including strong social stigma, poor understanding of autism, low social acceptance, and a cultural background that values averageness.

### Supplementary Information

**Additional file 1.** **Table S1.** Pearson correlations between all variables for the Japanese sample (N = 210). **Table S2.** Pearson correlations between all variables for the Japanese sample excluding straight liners (n = 204). **Table S3.** Hierarchical regression models predicting generalised anxiety (Model 1), depression (Model 2), social anxiety (Model 3), and well-being (Model 4) according to age, autistic traits, and social camouflage in the Japanese sample while excluding straight liners (n = 204). **Table S4.** Hierarchical regression models predicting generalised anxiety (Model 1a), depression (Model 2a), social anxiety (Model 3a), and well-being (Model 4a) according to age, sex, autistic traits, and social camouflage in the Japanese sample while excluding straight liners (N = 204). **Figure S1.** Scatter plots between standardized scores for generalized anxiety, depression, social anxiety, well-being, and social camouflage in the Japanese sample while excluding straight liners (N = 204).

**Additional file 2.** **Supplementary Data 1.** Analyzed dataset of responses to questionnaires by 210 autistic adults in Japan.

## Data Availability

The anonymised data set is available upon reasonable request from Fumiyo Oshima (c21ujsw35117c@faculty.gs.chiba-u.jp). The anonymised dataset of the sample from Japan is available in Additional file 2: Supplementary Data 1 (Data S1).

## Abbreviations

SD: Standard deviation
CAT-Q: Camouflaging autistic traits questionnaire
CAT-Q-J: Japanese version of the CAT-Q
LSAS-J: Liebowitz social anxiety scale
WEMWBS-J: Warwick–Edinburgh mental well-being scale
PHQ-9: Patient health questionnaire-9
GAD-7: Generalized anxiety disorder-7
