# Supplementary material for: The association between social camouflage and mental health among autistic people in Japan and the UK: a cross-cultural study
Source: Mol Autism. 2024 Jan 4;15:1. doi: 10.1186/s13229-023-00579-w (PMC10768303; doi:10.1186/s13229-023-00579-w)
Supplement: Supplementary file 1 — Additional file 1. Table S1. Pearson correlations between all variables for the Japanese sample (N = 210). Table S2. Pearson correlations between all variables for the Japanese sample excluding straight liners (n = 204). Table S3. Hierarchical regression models predicting generalised anxiety (Model 1), depression (Model 2), social anxiety (Model 3), and well-being (Model 4) according to age, autistic traits, and social camouflage in the Japanese sample while excluding straight liners (n = 204). Table S4. Hierarchical regression models predicting generalised anxiety (Model 1a), depression (Model 2a), social anxiety (Model 3a), and well-being (Model 4a) according to age, sex, autistic traits, and social camouflage in the Japanese sample while excluding straight liners (N = 204). Figure S1. Scatter plots between standardized scores for generalized anxiety, depression, social anxiety, well-being, and social camouflage in the Japanese sample while excluding straight liners (N = 204). [file 13229_2023_579_MOESM1_ESM.docx]

**Supplementary Materials**

**The association between social camouflage and mental health among autistic people in Japan and the UK: A cross-cultural study**

**Supplementary Tables**

**Table S1.** Pearson correlations between all variables for the Japanese sample (N = 210)

|  |  | **Age** | **CAT-Q** | **PHQ-9** | **GAD-7** | **LSAS** | **WEMWBS** |
| --- | --- | --- | --- | --- | --- | --- | --- |
| CAT-Q | r | -.267^**^ |  |  |  |  |  |
|  | p | < .001 |  |  |  |  |  |
| PHQ-9 | r | .050 | .176^*^ |  |  |  |  |
|  | p | .472 | .010 |  |  |  |  |
| GAD-7 | r | .033 | .144^*^ | .744^**^ |  |  |  |
|  | p | .640 | .039 | < .001 |  |  |  |
| LSAS | r | .034 | .187^**^ | .425^**^ | .503^**^ |  |  |
|  | p | .640 | .007 | < .001 | < .001 |  |  |
| WEMWBS | r | -.062 | .020 | -.684^**^ | -.593^**^ | -.477^**^ |  |
|  | p | .368 | .779 | < .001 | < .001 | < .001 |  |
| BAPQ | r | -.048 | .273^**^ | .484^**^ | .420^**^ | .590^**^ | -.524^**^ |
|  | p | .491 | < .001 | < .001 | < .001 | < .001 | < .001 |

BAPQ, Broad Autism Phenotype Questionnaire; CAT-Q, Camouflaging Autistic Traits Questionnaire; GAD-7, Generalized Anxiety Disorder Assessment; LSAS, Leibowitz Social Anxiety Scale; PHQ-9, Patient Health Questionnaire; WEMWBS, Warwick–Edinburgh Mental Well-being Scale.

**Table S2.** Pearson correlations between all variables for the Japanese sample excluding straight liners (n = 204)

|  |  | **Age** | **CAT-Q** | **PHQ-9** | **GAD-7** | **LSAS** | **WEMWBS** |
| --- | --- | --- | --- | --- | --- | --- | --- |
| CAT-Q | r | -.268^**^ |  |  |  |  |  |
|  | p | < .001 |  |  |  |  |  |
| PHQ-9 | r | .037 | .193^**^ |  |  |  |  |
|  | p | .597 | .006 |  |  |  |  |
| GAD-7 | r | .028 | .184^**^ | .769^**^ |  |  |  |
|  | p | .691 | .009 | < .001 |  |  |  |
| LSAS | r | .031 | .212^**^ | .444^**^ | .482^**^ |  |  |
|  | p | .680 | .004 | < .001 | < .001 |  |  |
| WEMWBS | r | -.071 | -.015 | -.710^**^ | -.588^**^ | -.476^**^ |  |
|  | p | .312 | .831 | < .001 | < .001 | < .001 |  |
| BAPQ | r | -.036 | .245^**^ | .496^**^ | .437^**^ | .597^**^ | -.546^**^ |
|  | p | .610 | < .001 | < .001 | < .001 | < .001 | < .001 |

BAPQ, Broad Autism Phenotype Questionnaire; CAT-Q, Camouflaging Autistic Traits Questionnaire; GAD-7, Generalized Anxiety Disorder Assessment; LSAS, Leibowitz Social Anxiety Scale; PHQ-9, Patient Health Questionnaire; WEMWBS, Warwick–Edinburgh Mental Well-being Scale.

**Table S3.** Hierarchical regression models predicting generalised anxiety (Model 1), depression (Model 2), social anxiety (Model 3), and well-being (Model 4) according to age, autistic traits, and social camouflage in the Japanese sample while excluding straight liners (n = 204)

|  | **Variable** | **β** | **p** | **95% confidence interval** | |
| --- | --- | --- | --- | --- | --- |
|  |  |  |  | **Lower** | **Upper** |
| Model 1 |  |  |  |  |  |
| (Generalised anxiety) | |  |  |  |  |
| Step 1 |  |  |  |  |  |
|  | Age | .044 | .490 | -.081 | .170 |
|  | BAPQ | .438 | < .001 | .313 | .563 |
| Step 2 |  |  |  |  |  |
|  | Age | .070 | .289 | -.060 | .201 |
|  | BAPQ | .414 | < .001 | .286 | .543 |
|  | CAT-Q | .102 | .138 | -.033 | .236 |
| Step 3 |  |  |  |  |  |
|  | Age | .074 | .261 | -.055 | .204 |
|  | BAPQ | .415 | < .001 | .287 | .543 |
|  | CAT-Q | .109 | .110 | -.025 | .244 |
|  | CAT-Q^2^ | .114 | .071 | -.010 | .238 |
| Model 2 |  |  |  |  |  |
| (Depression) |  |  |  |  |  |
| Step 1 |  |  |  |  |  |
|  | Age | .056 | .369 | -.066 | .177 |
|  | BAPQ | .498 | < .001 | .378 | .619 |
| Step 2 |  |  |  |  |  |
|  | Age | .081 | .206 | -.044 | .206 |
|  | BAPQ | .475 | < .001 | .351 | .599 |
|  | CAT-Q | .098 | .134 | -.030 | .226 |
| Step 3 |  |  |  |  |  |
|  | Age | .084 | .187 | -.041 | .209 |
|  | BAPQ | .476 | < .001 | .352 | .599 |
|  | CAT-Q | .104 | .109 | -.023 | .232 |
|  | CAT-Q^2^ | .097 | .112 | -.022 | .216 |
| Model 3 |  |  |  |  |  |
| (Social anxiety) | |  |  |  |  |
| Step 1 |  |  |  |  |  |
|  | Age | .053 | .424 | -.079 | .186 |
|  | BAPQ | .599 | < .001 | .487 | .712 |
| Step 2 |  |  |  |  |  |
|  | Age | .077 | .258 | -.058 | .211 |
|  | BAPQ | .578 | < .001 | .460 | .696 |
|  | CAT-Q | .090 | .159 | -.036 | .216 |
| Step 3 |  |  |  |  |  |
|  | Age | .078 | .253 | -.057 | .212 |
|  | BAPQ | .578 | < .001 | .460 | .696 |
|  | CAT-Q | .092 | .154 | -.035 | .219 |
|  | CAT-Q^2^ | .027 | .641 | -.086 | .140 |
| Model 4 |  |  |  |  |  |
| (Well-being) |  |  |  |  |  |
| Step 1 |  |  |  |  |  |
|  | Age | -.091 | .123 | -.207 | .025 |
|  | BAPQ | -.549 | < .001 | -.667 | -.431 |
| Step 2 |  |  |  |  |  |
|  | Age | -.063 | .304 | -.183 | .057 |
|  | BAPQ | -.575 | < .001 | -.696 | -.453 |
|  | CAT-Q | .109 | .084 | -.014 | .232 |
| Step 3 |  |  |  |  |  |
|  | Age | -.066 | .281 | -.185 | .054 |
|  | BAPQ | -.575 | < .001 | -.696 | -.454 |
|  | CAT-Q | .103 | .101 | -.020 | .226 |
|  | CAT-Q^2^ | -.089 | .130 | -.203 | .026 |

BAPQ, Broad Autism Phenotype Questionnaire; CAT-Q, Camouflaging Autistic Traits Questionnaire; β, standardised beta. The quadratic term of standardised social camouflage scores (CAT-Q^2^) is added in Step 3.

**Table S4.** Hierarchical regression models predicting generalised anxiety (Model 1a), depression (Model 2a), social anxiety (Model 3a), and well-being (Model 4a) according to age, sex, autistic traits, and social camouflage in the Japanese sample while excluding straight liners (N = 204)

|  | **Variable** | **β** | **p** | **95% confidence interval** | |
| --- | --- | --- | --- | --- | --- |
|  |  |  |  | **Lower** | **Upper** |
| Model 1 |  |  |  |  |  |
| (Generalised anxiety) | |  |  |  |  |
| Step 1 |  |  |  |  |  |
|  | Age | .089 | .175 | -.040 | .217 |
|  | Sex | .182 | .005 | .056 | .309 |
|  | BAPQ | .403 | < .001 | .276 | .530 |
|  | CAT-Q | .068 | .319 | -.066 | .202 |
| Step 2 |  |  |  |  |  |
|  | Age | .089 | .177 | -.040 | .218 |
|  | Sex | .182 | .005 | .055 | .309 |
|  | BAPQ | .404 | < .001 | .276 | .531 |
|  | CAT-Q | .068 | .325 | -.067 | .202 |
|  | CAT-Q*Sex | .004 | .956 | -.121 | .128 |
| Model 2 |  |  |  |  |  |
| (Depression) |  |  |  |  |  |
| Step 1 |  |  |  |  |  |
|  | Age | .100 | .115 | -.024 | .223 |
|  | Sex | .184 | .003 | .061 | .306 |
|  | BAPQ | .464 | < .001 | .343 | .586 |
|  | CAT-Q | .064 | .325 | -.063 | .191 |
| Step 2 |  |  |  |  |  |
|  | Age | .099 | .117 | -.025 | .223 |
|  | Sex | .183 | .003 | .060 | .306 |
|  | BAPQ | .465 | < .001 | .343 | .588 |
|  | CAT-Q | .062 | .340 | -.066 | .191 |
|  | CAT-Q*Sex | .013 | .832 | -.106 | .132 |
| Model 3 |  |  |  |  |  |
| (Social anxiety) | |  |  |  |  |
| Step 1 |  |  |  |  |  |
|  | Age | .078 | .261 | -.060 | .215 |
|  | Sex | .012 | .850 | -.110 | .133 |
|  | BAPQ | .578 | < .001 | .460 | .696 |
|  | CAT-Q | .088 | .179 | -.041 | .217 |
| Step 2 |  |  |  |  |  |
|  | Age | .079 | .253 | -.058 | .216 |
|  | Sex | .014 | .819 | -.107 | .136 |
|  | BAPQ | .574 | < .001 | .456 | .693 |
|  | CAT-Q | .093 | .160 | -.037 | .223 |
|  | CAT-Q*Sex | -.042 | .475 | -.155 | .072 |
| Model ４ |  |  |  |  |  |
| (Well-being) |  |  |  |  |  |
|  | Age | -.073 | .233 | -.193 | .047 |
|  | Sex | -.101 | .097 | -.220 | .018 |
|  | BAPQ | -.569 | < .001 | -.689 | -.448 |
|  | CAT-Q | .128 | .045 | .003 | .252 |
| Step 2 |  |  |  |  |  |
|  | Age | -.071 | .248 | -.191 | .049 |
|  | Sex | -.096 | .113 | -.216 | .023 |
|  | BAPQ | -.574 | < .001 | -.695 | -.454 |
|  | CAT-Q | .136 | .032 | .011 | .261 |
|  | CAT-Q*Sex | -.076 | .196 | -.191 | .039 |

BAPQ, Broad Autism Phenotype Questionnaire; CAT-Q, Camouflaging Autistic Traits Questionnaire.

**Supplementary Figure**


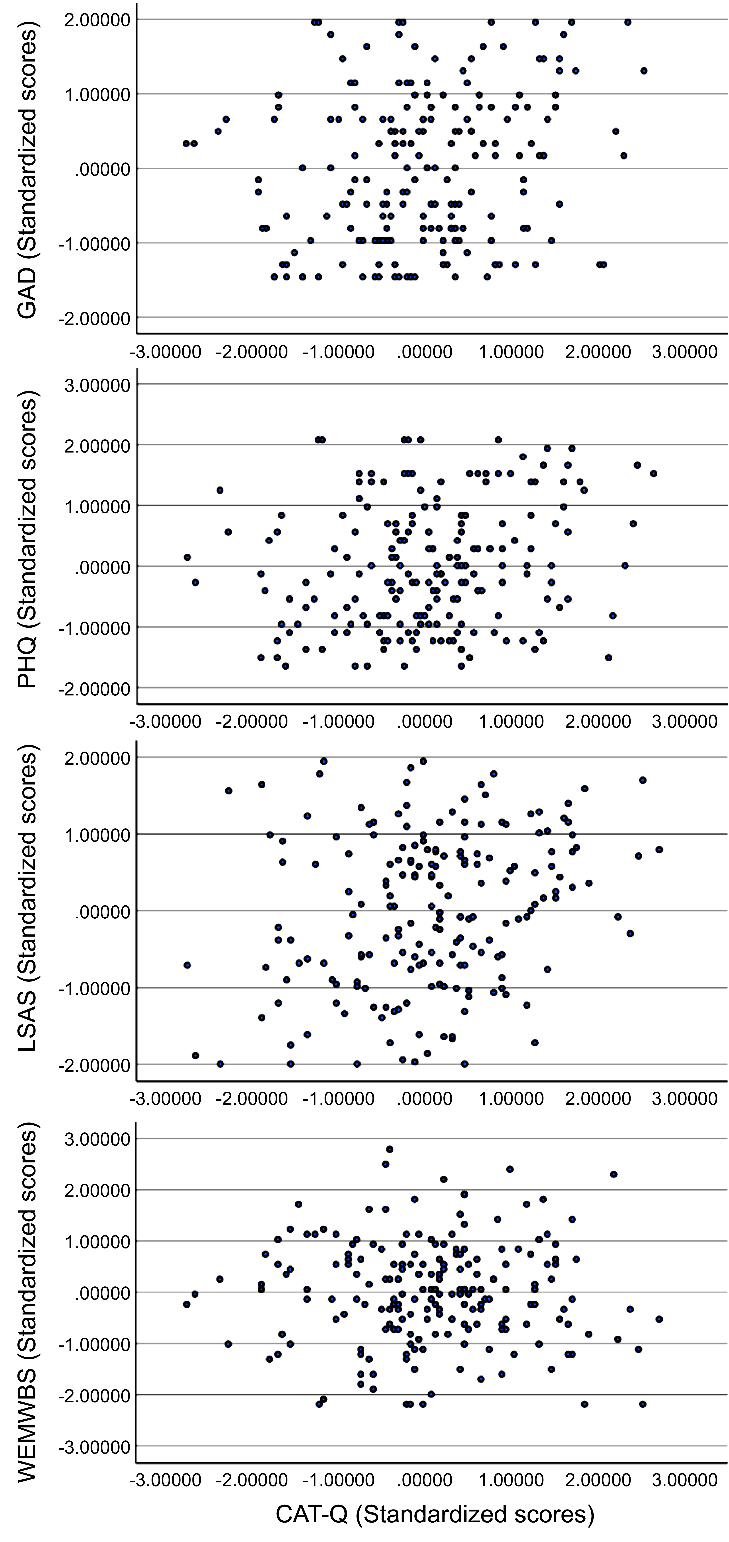


**Figure S1**. Scatter plots between standardized scores for generalized anxiety, depression, social anxiety, well-being, and social camouflage in the Japanese sample while excluding straight liners (N = 204)
